# Insect Leaf-Chewing Damage Tracks Herbivore Richness in Modern and Ancient Forests

**DOI:** 10.1371/journal.pone.0094950

**Published:** 2014-05-02

**Authors:** Mónica R. Carvalho, Peter Wilf, Héctor Barrios, Donald M. Windsor, Ellen D. Currano, Conrad C. Labandeira, Carlos A. Jaramillo

**Affiliations:** 1 Department of Geosciences, Pennsylvania State University, University Park, Pennsylvania, United States of America; 2 Department of Plant Biology, Mann Library Building, Cornell University, Ithaca, New York, United States of America; 3 Programa de Maestría en Entomología, Universidad de Panamá, Provincia de Panamá, Panamá; 4 Smithsonian Tropical Research Institute, Balboa, Ancón, Republic of Panamá; 5 Department of Geology and Environmental Earth Science, Miami University, Oxford, Ohio, United States of America; 6 Department of Paleobiology, Smithsonian Institution, Washington, DC, United States of America; 7 Department of Entomology and BEES Program, University of Maryland, College Park, Maryland, United States of America; 8 Smithsonian Tropical Research Institute, Balboa, Ancón, Republic of Panamá; Institut National de la Recherche Agronomique (INRA), France

## Abstract

The fossil record demonstrates that past climate changes and extinctions significantly affected the diversity of insect leaf-feeding damage, implying that the richness of damage types reflects that of the unsampled damage makers, and that the two are correlated through time. However, this relationship has not been quantified for living leaf-chewing insects, whose richness and mouthpart convergence have obscured their value for understanding past and present herbivore diversity. We hypothesized that the correlation of leaf-chewing damage types (DTs) and damage maker richness is directly observable in living forests. Using canopy access cranes at two lowland tropical rainforest sites in Panamá to survey 24 host-plant species, we found significant correlations between the numbers of leaf chewing insect species collected and the numbers of DTs observed to be made by the same species in feeding experiments, strongly supporting our hypothesis. Damage type richness was largely driven by insect species that make multiple DTs. Also, the rank-order abundances of DTs recorded at the Panamá sites and across a set of latest Cretaceous to middle Eocene fossil floras were highly correlated, indicating remarkable consistency of feeding-mode distributions through time. Most fossil and modern host-plant pairs displayed high similarity indices for their leaf-chewing DTs, but informative differences and trends in fossil damage composition became apparent when endophytic damage was included. Our results greatly expand the potential of insect-mediated leaf damage for interpreting insect herbivore richness and compositional heterogeneity from fossil floras and, equally promisingly, in living forests.

## Introduction

The plant-insect system's response to climate change and extinction in deep time has been studied using tens of thousands of fossil leaves with insect-feeding damage as primary data [1]–[11]. Insect-mediated damage types (DTs) in the leaf-fossil record consistently show that DT richness (DTR) positively tracks paleotemperatures [1], [5], [9], and severe and sustained drops in DTR in the western USA remain the only spatially and temporally well-constrained data that show the fate of insects across the end-Cretaceous extinction [1]–[4], [12]. These paleontological results are consistent among many basins and time periods, making them highly relevant as context for current climatic and other anthropogenic change [13]; they are observed both for host-specific endophytic DTs (leaf mines and galls) and for total damage, including piercing and external leaf-chewing damage. The recurring correlations between endophytic and total DTR among fossil host-plant species within sites, and among bulk samples, strongly suggests that important, underlying biological mechanisms determine total DTR, and that there is a quantitative relationship between DTR and the richness of the corresponding but unpreserved damage makers (insect richness, hereafter IR).

**Figure 1:**
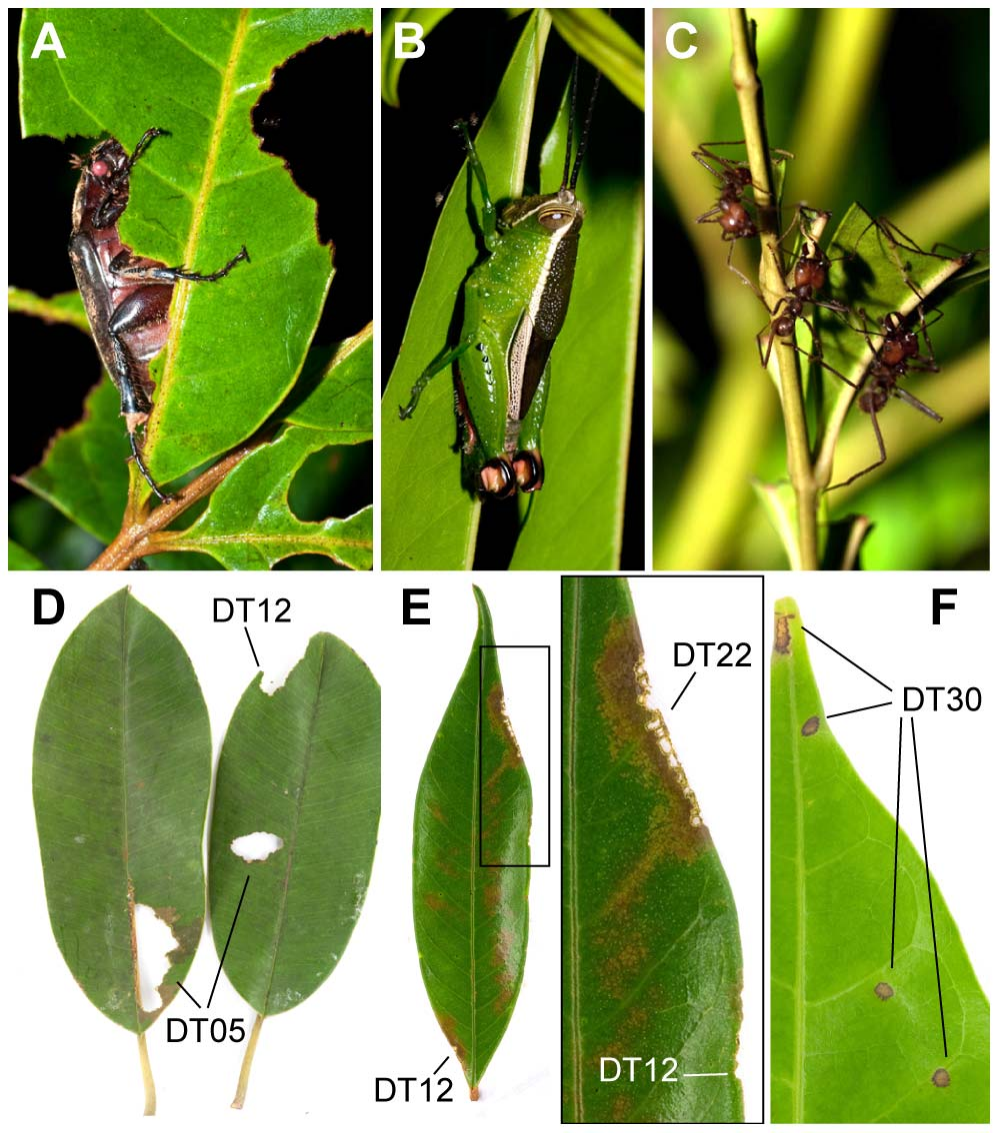
Selected leaf-chewing insects collected from two Panamanian forests, and induced external leaf damage resulting from insect feeding experiments. (*A*) *Phyllophaga* sp. 2 (Coleoptera: Scarabaeidae) observed inducing margin feeding DT12 and DT14 on *Tapirira guianensis* Aubl. (Anacardiaceae). (*B*) Tettigoniidae sp.4. (Orthoptera) on leaves of *Guatteria dumetorum* R. E. Fr (Annonaceae). (*C*) *Atta* sp.1 (Hymenoptera: Formicidae) on leaflets of *Jacaranda copaia* (Aubl.) D. Don (Bignoniaceae) causing margin feeding DT13. (*D*) Hole and margin feeding DT05, DT12 on leaves of *Manilkara bidentata* (A. DC.) A. Chev. (Sapotaceae) induced by Tettigoniidae sp.5 (Orthoptera). Sample 09-216. (*E*) Margin feeding DT12 and skeletonization DT22 on leaves of *Vochysia ferruginea* Mart. (Vochysiaceae) induced by multidamaging species Cryptocephalinae sp.1 (Coleoptera: Chrysomelidae). Sample 09-131. (*F*) Surface feeding damage DT30 on leaf of *T. guianensis*, inflicted by monodamaging species *Homeolabus analis* Illiger (Coleoptera: Attelabidae). Sample 09-135.

For most extant endophytic feeding, it is well known that many insect species make single, distinct DTs on single host species at a site [1], [5], [9], [14], [15]. However, external feeders (leaf-chewers; Figs. 1A–C) are often less host-specialized, produce the most abundant and diverse leaf damage (Figs. 1D–F), and are thought to represent the great majority of herbivore species [16]. External damage is induced by mandibulate insects, which include species of a large number of distantly related taxa that feed on leaves through life cycle stages [17], [18], and also includes insect-induced damage that is not caused by direct feeding, such as vein and leaf cutting by various groups [19]. The greatest diversity of leaf-chewers lies in Coleoptera (beetles), but other significant groups include Orthoptera (grasshoppers), Phasmida (stick insects), and Lepidoptera (caterpillars). Damage types related to leaf chewing are numerous today and in the fossil record [4], reflecting the many insect species and ontogenetic stages that produce them (larvae, nymphs, adults). Due to widespread mouthpart and behavioral convergence, only in few cases do external feeding DTs found in fossils appear to be assignable to specific taxonomic groups [20], [21], whereas most DTs that are confidently associated to a particular culprit correspond to endophagous feeding such as leaf mining [22].

Overall, the great abundance and diversity of leaf-chewing insects, the general lack of knowledge regarding their specific feeding behaviors, their convergence on mouthpart features [23], and the prodigious damaging abilities of certain species [24], [25], all indicate that a quantitative link between external DTR and the number of culprit insect species (insect richness – IR) may not exist, or be extremely weak. However, this relationship has not been systematically addressed, and the consistent correlation in the fossil record of total and endophytic DTR strongly suggests that it may be significant [3], [9]. The few actualistic studies of insect-damaged leaves to date, while helpful, did not include insect sampling [26], [27]. However, some investigations demonstrated instances of DTR following a latitudinal gradient in the eastern USA [28] and responding to biotic host plant conditions such as seedling abundance and proximity to closely related neighbors [29].

The external-feeding fraction of insect damage has great potential for improving understanding of ancient and extant herbivore communities because of its ubiquitous nature and the large sample sizes available. For example, inventorying and monitoring suites of leaf-damage types in living forests potentially offers opportunities for assessing insect richness, or detecting changes in insect composition along climatic, edaphic, or temporal gradients, much more rapidly than is possible via standard collecting of insects.

Here, for the first time, we test directly for a quantitative relationship between the numbers of leaf-chewing insect species and the DT richness induced by the same sampled insects under observation, among single host-plant species. In summary (see Materials and Methods for details), testing this relationship was done by observing all feeding-related DTs made by individuals of each insect species found in the canopies of 24 species of dominant canopy angiosperm trees and lianas, at equivalent sampling intensity among host plants. Canopy leaves and insects were accessed using research cranes located at two lowland tropical rainforest sites in Panamá. We collected leaf-chewing insects and quantified the number of distinct leaf damage types that these individuals produced when fed with fresh, undamaged leaves from their host plants in feeding experiments. We then compared our results to fossil leaf damage from a variety of well preserved, sampled, and dated fossil angiosperm assemblages from the latest Cretaceous through middle Eocene of North and South America to assess how our approach can be used to improve understanding of insect-damage richness and composition in fossil floras.

## Materials and Methods

### Study Sites and Sampling Permits

All collection procedures complied with local and international regulations, and all vouchered material remained in Panamanian territory. Collection permits were granted by the Autoridad Nacional del Ambiente (ANAM). Fieldwork was conducted at the canopy access crane sites of the Smithsonian Tropical Research Institute in the Republic of Panamá [30]. One crane is located near the Pacific coast in the seasonally dry forest at Parque Natural Metropolitano (PNM: 8°59N, 79°33W, elevation 50 m), in Panamá City, the other in the everwet rainforest at Área Protegida de San Lorenzo, near Colón on the Caribbean coast (APSL: 9°17′N, 79°58′W, elevation 25 m). Both sites belong to the national system for protected areas of Panamá.

### Data Collection

Twelve dominant tree and liana species were selected at each site that had greatest canopy coverage in the crane plots, as estimated by basal stem area coverage for trees (data available at www.stri.org) and by visual inspection for lianas (Table S1). Canopy branches of the selected plant species were randomly and extensively inspected, with near-equal time allotted to each host species, for insect herbivores during the new moon and adjacent nights of the first three months of the wet seasons of 2008 and 2009. Between sunset and midnight, we observed and collected all insects in the orders Coleoptera, Hymenoptera, Lepidoptera, and Orthoptera showing leaf-chewing behavior. Although leaf damage induced by leaf-cutting ants and vein-cutting longhorn beetles is not directly caused by plant consumption, it was included because such damage is related to feeding behavior. Insects were collected by hand capture or branch-beating and placed in bags, and later subjected to feeding experiments using undamaged fresh leaves from the same branches at the field station of APSL or at the facilities of the STRI Center for Tropical Paleoecology and Archaeology in Panamá City.

Adult stage insects were allowed to feed over 2–3 days, and immatures were reared to full maturity when possible to observe ontogenetic variation in feeding style. Partially eaten leaves were photographed, and the induced leaf-chewing damage was classified into 43 discrete DTs (see Fig. S1 following the *Guide to Insect (and other) Damage Types on Compressed Plant Fossils* [4], which describes a method widely used on fossil leaf assemblages [1]–[5], [7]–[10]. Fourteen external-feeding DTs recorded in this study did not match any described fossil DTs of ref. [4] and were recorded as “new DTs” (NDT; see Fig. S2 and Table S2 for descriptions). Feeding insects were mounted and identified to species level when possible, or otherwise split into identifiable morphospecies under a family or genus name (Dataset S1). A total of 276 samples (each an insect plus its damaged leaves) containing 156 species of leaf-chewing insects were collected and deposited together in the STRI Insect Collection under the collection code ‘LDP’ (Leaf Damage Project), comprising, to our knowledge, the only vouchered collection of diverse, identified insects and their feeding damage on leaves of identified plant hosts.

### Quantitative Analyses

**Figure 2:**
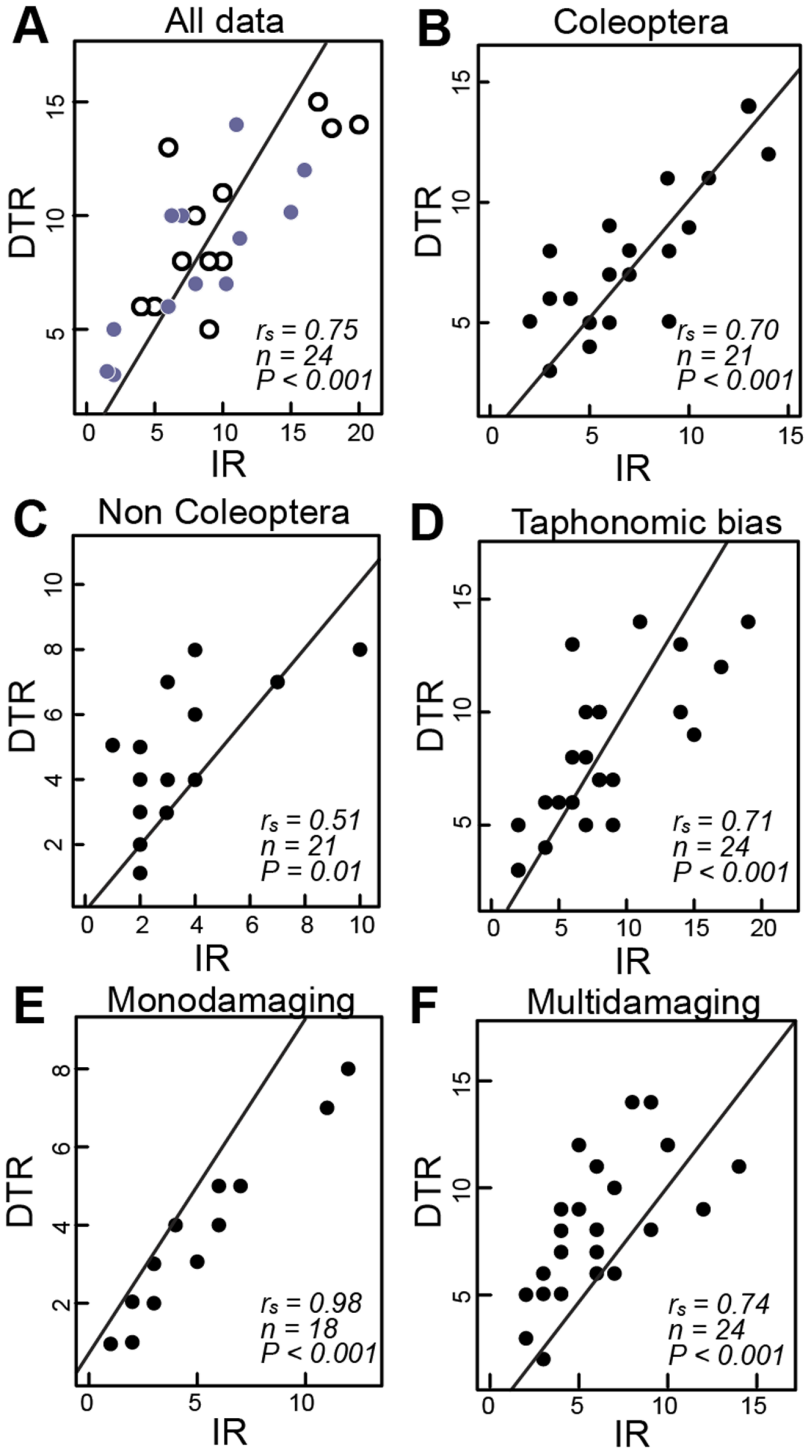
Correlations of insect and induced leaf-damage richness in two Panamanian forests. Spearman's correlations (r_s_) of the number of insect species (Insect richness – IR) vs. number of leaf-chewing damage types (damage type richness – DTR) observed across 24 tropical host plant species, plotted against 1∶1 lines, include: (*A*) Total numbers of collected leaf-chewing insect species per host plant at Área Protegida de San Lorenzo (Gray, solid circles) and Parque Natural Metropolitano (black, hollow circles). (*B*) IR-DTR correlation for coleopteran species. (*C*) IR-DTR correlation for non-coleopteran species. (*D*) IR-DTR correlation after filtering out leaf damage that is unlikely to be preserved in fossil assemblages. (*E*–*F*). Separate IR-DTR correlations for insect species that induced one (monodamaging, E) or multiple (multidamaging, F) DTs when feeding. For (*A*–*F*), each data point is one host-plant species at one site; all data plot with a shallower slope than 1∶1, as expected from convergence.

All statistical analyses were performed using R version 2.15 [31]. At each site, we compared the number of collected insect species (insect richness, IR) and insect families on each host-plant species, to the number of different DTs (external damage type richness, DTR) that they produced in the feeding experiments. Because no statistical differences were observed between the IR-DTR correlations at the Área Protegida de San Lorenzo and Parque Natural Metropolitano, the data from both sites were pooled in further analyses. Total numbers of insect species were correlated with their induced DT richness and their coefficient of determination is reported (Spearman's rho, r_s_; Figs. 2A–E; Table S3). These correlations were also tested with values subsampled to 20 individuals per host-plant species (Table S3).

Potential biases in comparing leaf damage between living and fossil assemblages were addressed by applying a binary “good-bad fossil” score for potential preservation and identification to each DT occurrence, presumptively suggesting a hypothetical, typical fossil leaf assemblage. This was done using our group's extensive previous experience with the insect-damage fossil record and knowledge of typical biases associated with leaf-compression fossils that have insect damage. Scoring was based on the relative probability that a particular DT on a particular leaf would become fossilized and ultimately be identified. Factors taken into consideration were wind and water-mediated transportation, deposition, burial, and other taphonomic processes such as the likelihood of preservation based on the conspicuousness and size of the damaged area. These criteria are relevant for leaf fossil assemblages that typically do not preserve delicate structures such as fine vein stringers, excisions, and epidermal tissue flaps. For testing a “biased preservation” correlation (Fig. 2D), the damage types labeled as unlikely to fossilize and their inflicting insects were removed from the dataset, and the remaining insect-damage pairs were used.

The relative frequency of DTs recorded in the feeding experiments was compared to relative abundance of DTs observed in the fossil assemblages. Damage types were ranked according to the corresponding number of culprit insect species found in the feeding experiments and were correlated to external DT rank-order at each of the fossil sites, as established from the averaged abundance of DTs observed in 500 randomly selected fossil leaves, subsampled 1000 times. Because fossil leaf assemblages reflect much larger areas and temporal scales, their corresponding leaf damage is likely to include the effect of additional factors such as herbivore population densities, leaf lifespan and taphonomic filters, among others. The relative abundances of DTs are not strictly comparable to the frequencies of DTs recorded in feeding experiments, and thus no further statistical comparisons were performed.

For the extant plots, host-plant pairwise similarity was estimated based on insect species composition and DT composition using the Chao-Sørensen index, which considers the probability of unobserved species (or here, DTs) based on frequency [32]. At each fossil site, host-plant pairwise similarity of external DTs was also computed using the Chao-Sørensen index after filtering for host plant species with collections of at least 25 leaves. The index was computed from the mean vectors of the DTs in 25 leaves from each species, subsampled 1000 times each and rounded up to the nearest integer. These observed pairwise similarities were compared to the null expectation that DTs are equally distributed across host-plant species, and their probability of occurrence on any given leaf follows the observed relative abundance of each DT on 500 randomly selected leaves at each site (see above). The pairwise similarities expected under this null hypothesis for each site were estimated by computing pairwise similarity indices from 25 leaves per host-plant species, considering that the probability of occurrence for any given DT on any given leaf was the same across all host-plant species, and was based on relative abundance. This was repeated 1000 times to create a distribution for our null hypothesis.

The same pairwise similarity approach was taken for comparing total damage, including leaf mining, piercing, and galling, across host plant species (ovipositional damage was excluded from these data). Similarities of total leaf damage were estimated using the Sørensen coefficient because this statistic is based on the total numbers of shared and unshared elements, and it is therefore not biased by the vast abundance of external feeding damage, compared to endophytic feeding damage, in the fossil assemblages. Similarity of total damage is reported as 1-Sørensen coefficient.

### Fossil Sites

Fossil floras with insect damage used here for comparison, and which largely motivated this study, came from northern Colombia, southern Argentina, and the Great Plains and Rocky Mountain regions of the USA. Their leaf-damage data were previously published and vouchered as cited below. The Colombian sample is the mid-Paleocene Cerrejón coal-swamp flora of Guajira, Colombia, representing the oldest known Neotropical rainforest and in that sense the most similar site to the extant Panamá plots; this flora is characterized by abundant and moderately diverse, but almost entirely external insect damage as reported in ref. [33]. The Argentine site is the early Eocene, volcanic-lacustrine Laguna del Hunco flora from Chubut, Patagonia, Argentina, which represents a hyperdiverse Gondwanic rainforest ecosystem [34], [35] with abundant and diverse insect damage [34], as well as novel insect body fossils [36], [37] that cannot be linked to the damage. The data used are from [34], all quarries pooled. A set of six sites comes from four latest Cretaceous and two early Paleocene horizons of alluvial strata in the Williston and Powder River basins of southwestern North Dakota and southeastern Montana, USA. These floras record regional extinction and recovery of the plant-insect system across the terminal Cretaceous event [2], [3], [12], [38], [39]. They include rich Cretaceous floras with generally high DTR, an immediate post-extinction “disaster flora” with minimal DTR (Pyramid Butte) and the bizarre “ecologically decoupled” assemblage of high DTR (especially of mining) and low floral diversity from the early Paleocene Mexican Hat site [3]. Damage data from these sites are as reported in [3]. A set of four late Paleocene and five early Eocene sites comes from alluvial strata of the Bighorn Basin, northwestern Wyoming, USA, recording a tight response of DTR to a series of pulsed and sustained warming and cooling events as reported in [5], [7], [9], namely gradual late Paleocene warming, a major pulse of warming at the Paleocene-Eocene Thermal Maximum, an early Eocene cool period, and renewed warming to the Early Eocene Climatic Optimum. Even though leaf damage, and particularly leaf galling, can be attributed to other non-insect arthropods (e.g. mites), external feeding damage in fossil leaves from these studies was attributed to mandibulate insects based on the recurrence of subsurface mouthpart marks, avoidance of indurated leaf structures (cell walls, lignified tissues), and reaction rims. Although it is possible that non-arthropod invertebrates such as pulmonate snails and slugs, may have inflicted some of the observed leaf-chewing damage, their radulae leave marks that cannot be confused with those of mandibulate arthropods [40].

## Results

We collected 276 adults and immature leaf-chewing insects of 156 species: 96 coleopterans, 38 orthopterans, 11 lepidopterans, nine phasmids, and two hymenopterans. The number of herbivore species collected on any particular host plant species varied between two and 20, while 31 insect species (ca. 20% of the total) were found on more than one host species. In each feeding experiment, herbivores left single or multiple DTs as feeding traces and were correspondingly categorized as “monodamagers” or “multidamagers” (see Dataset S1).

The number of leaf-chewing insect species (insect richness, IR) collected from each host-plant species showed a robust, positive correlation with the number of DTs (damage type richness, DTR) recorded for the same host-plant species (Fig. 2A). This correlation was also significant at the insect-family level (Spearman's r_s_ = 0.79, *P*<0.001; Table S3); when considering coleopteran and non-coleopteran species separately (Figs. 2B,C); and after removing all DTs (and corresponding culprits) that had low fossilization potential (Fig. 2D; see Materials and Methods). Also, these correlations did not differ significantly between the two sampling sites (Z-test *P* = 0.92). The numbers of monodamaging and multidamaging insects collected on each host-plant species were each correlated significantly to their numbers of induced DTs (Figs. 2E,F; see Tables S1 and S3).

The rank-order of DTs, ordinated according to the number of insect species recording each DT, did not differ between the two modern sites (Mann-Whitney test *P* = 0.37), indicating that the most commonly shared DTs across herbivores are the same at both forest plots. Figure S3 summarizes the relative number of insect species inflicting each DT. We then tested for correspondence between the relative abundances of DTs at each fossil site (see Fig. S4) and the relative proportions of collected living herbivore species sharing each DT. Spearman's rank-order correlations between each fossil site and the pooled extant sites were all highly significant (Table S4), indicating that the ranking of DTs in the Cretaceous and Paleogene floras is broadly similar to that of DTs most commonly induced by our living insect species.

**Figure 3:**
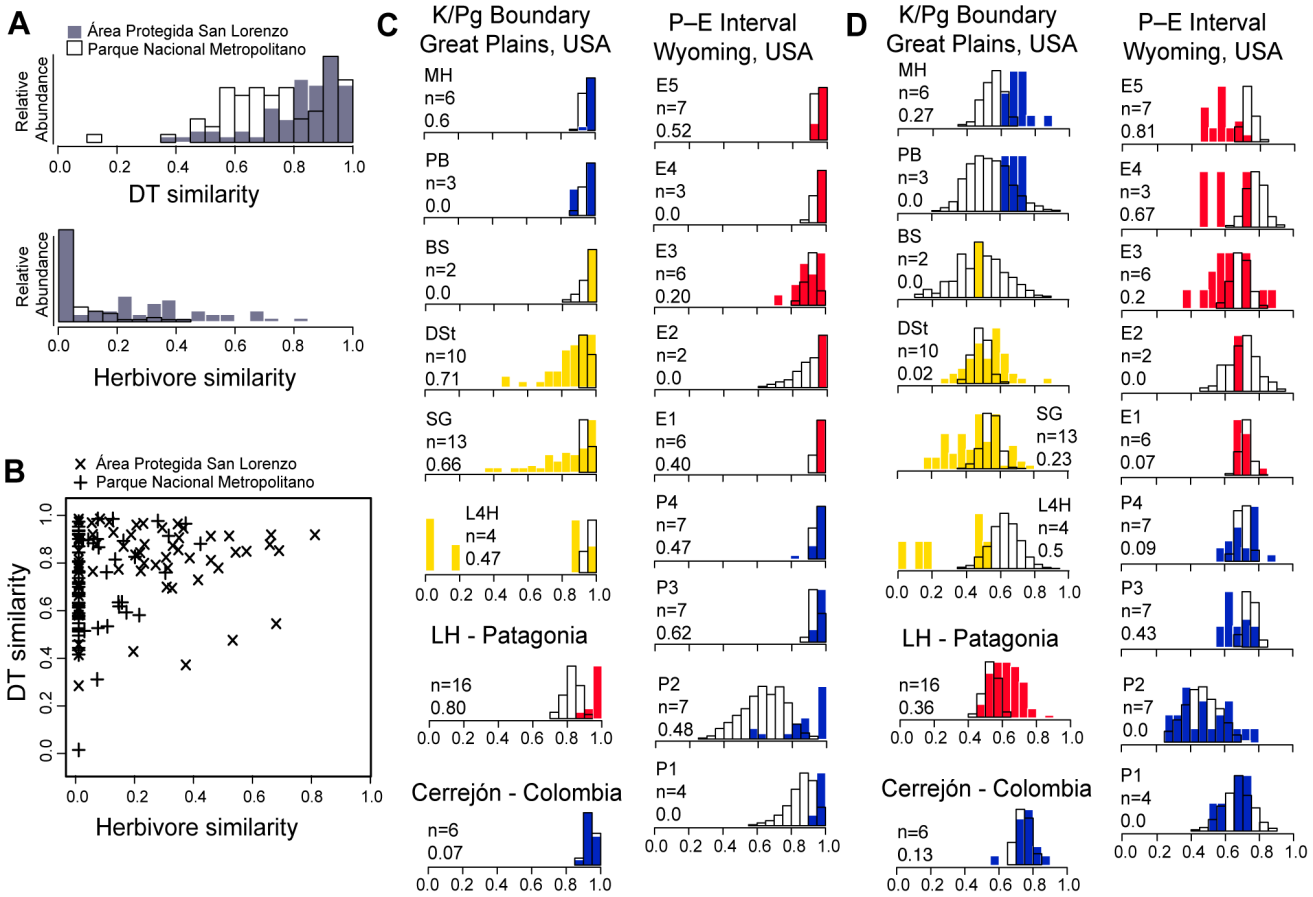
Pairwise host-plant similarities based on herbivores and leaf damage in living forests, and on leaf damage in fossil forests. (*A*) Inverse relations between the distributions of pairwise similarities of leaf damage (DT) and herbivore species, estimated using the Chao-Sørensen index, for host-plant species pairs at the Área Protegida de San Lorenzo (gray bars) and Parque Natural Metropolitano (hollow bars), Panamá (Dataset S1). Vertical axis indicates relative abundance of species pairs showing a given similarity index (horizontal axis). Similarity of DTs between host-plant pairs is much higher than similarity of herbivores, as expected from convergence. (*B*) Plant-species pairwise similarities of herbivores and leaf damage, from the Panamá crane sites (same as in *A*). (*C*) Skewed distributions of host-plant pairwise similarities of leaf-chewing damage at fossil sites (see Materials and Methods), estimated for (n) species at each fossil site, using the Chao-Sørensen index (Dataset S1). Axes are similar to those in (*A*). Yellow  =  Late Cretaceous, blue  =  Paleocene, and red  =  Eocene age. Hollow bars represent distributions of the null expectation, and numbers indicate how many host-plant species (n) and the proportion of observed pairwise estimates that fall outside the null distribution range. (*D*) Distributions of host-plant pairwise similarities of total leaf damage (i.e., including endophytic, largely specialized feeding such as mines and galls) at fossil sites as in (C), estimated as 1-Sørensen coefficient (Dataset S1). There is greatly increased distinctiveness in damage type composition between host plants when endophytic feeding is considered when compared to (*C*).

For plant species at both canopy-crane sites, host-plant pairwise similarities of herbivore and leaf-chewing DT composition (Chao-Sørensen index) only matched well in two scenarios (Fig. 3A,B): values indicating low similarity of DTs across host-plant species were only observed for host species-pairs with low similarity of insects, and conversely, high similarity of feeding insects was reflected in high similarity of DTs. However, high DT similarity of leaf-chewing was not informative in terms of herbivore similarity (Fig. 3A,B). Likewise, host-plant pairwise DT similarities for each fossil site (Fig. 3C) showed that nearly all species pairs were very similar in leaf-chewing DT composition (Chao-Sørensen index >0.8), thus being uninformative in terms of herbivore similarity in the fossil record. However, species pairwise differences became apparent when estimated using the presence or absence of all leaf-damage (Sørensen index), including leaf mining, galling and other endophagous DTs (Fig. 3D), instead of solely using leaf chewing (external) damage (Fig. 3C).

## Discussion

### Damage-Type Richness Correlates with Culprit Richness

The robust correlations found between IR and DTR in two tropical forest plots (Fig. 2A) indicate that the number of leaf-chewing insect species is indeed reflected in the number of external DTs observed on a host-plant species. This relationship is consistent with the recurrent similarity in fossil assemblages between endophytic and external damage that suggested both categories of damage were related to actual diversity of their damage makers. Coleopterans comprised over half the collected insect species, yet the number of coleopteran and non-coleopteran species were each separately correlated to their induced external damage (Figs. 2B,C), showing that the link between IR and external-DTR is not solely driven by beetles. Rather, our observations suggest that the relation is a common and intrinsic pattern for all chewing insects, presumably driven by repeated selection through time by factors such as ecological partitioning by both plants and insects, competition, other interspecies interactions, and abiotic forcing.

A number of DTs were common to almost all collected insect species at both sites (see Figs. 1D, E), whether they were monodamagers or multidamagers. These included hole feeding types DT02, DT04, and DT05, and margin feeding types DT12, DT13, and DT14. Based on the number of herbivore species, we categorized a fraction of DTs as ‘rare’ if < = 10% of the total collected herbivore species produced them (Dataset S1). We found that these rare DTs were usually produced by multidamager species that also recorded all the common DTs, which were also made by the monodamagers. These rare DTs are major contributors to DTR, and thus we conclude that DTR is intimately related to the presence and diversity of multidamaging insects.

Multidamaging ability was found across all taxonomic orders and guilds collected. For example, changes in body size, and consequently in mandible size, related to moulting of coleopteran and lepidopteran larvae are reflected in the strength and ability to gain purchase and excise foliar tissues [17], and they allow for feeding behavior that progresses with development from scraping by more gracile mouthparts, then skeletonizing, and eventually to leaf excision with increasingly robust mouthpart structures [18]. Adult stages of coleopterans and orthopterans were also observed to induce more than one DT, including the production of various types of hole and margin feeding marks (Fig. 1D), despite the frequent observation of margin feeding being characteristic of orthopteroids [41]. Even species of leaf-cutting ants (Fig. 1C) recorded multiple DTs. Despite the distinctive feeding behavior of this group [42], their recorded damage followed the same pattern observed for most chewing insects, in which rare DTs, specifically associated in this case with leaf excision by ant mandibles (NDT05; Fig. S2 and Table S2), were also associated with other DTs shared across most insect species (margin feeding types DT12, DT13, DT14).

### Importance for the Fossil Record

Our results strongly relate leaf-chewing insect species to their recorded leaf damage, despite mouthpart convergence and regardless of common preservation or identification biases, and thus provide a considerably advanced framework for interpreting leaf-chewing damage. First and foremost, the frequent and societally relevant observations from the fossil record of decreasing DTR with cooling and after extinctions, and of increasing DTR during warming events and post-extinction recoveries [1]–[3], [5], [8]–[10], [12], can now be related to changes in actual insect richness on a sound observational basis and not only through deductive logic. External feeding should no longer be regarded as too “noisy” to be informative, and analyzing external damage along with endophytic damage, as usually practiced, serves to increase the signal to noise ratio much further.

We emphasize, however, that until more extant sites are studied in this way, our findings can only support qualitative or relative, not absolute, inferences of differences in insect richness. Also, the existence of the IR-DTR correlation is demonstrated here among host plants that share many DTs, and it thus falls below the 1∶1 line as expected because of convergence (e.g., Fig. 2A–F); the existence of a correlation should not be confused with any sort of general absolute equivalence between external DTs and insect species.

Removing damage types with low preservation potential did not obscure the quantitative relation between leaf damage and culprit richness in Panamá (Fig. 2D). The consistent correlation between IR and DTR indicates that despite the presumed loss of some DT data, at least a relative signal for insect richness can be confidently assessed from leaf assemblages, fossil or living. In most cases, DTs that we considered prone to taphonomic loss were recorded by multidamaging culprits that concurrently inflicted other, less delicate types of damage (Dataset S1). Thus, despite the loss of fragile or poorly identifiable DTs, most culprit insect species are still represented in the remaining DT pool.

It is striking that the rank-ordered abundances of external DTs recorded from each of the 17 leaf-fossil assemblages correlate at high significance levels with the pooled data from the two Panamá crane sites (Dataset S1). This finding shows a clear and previously unknown consistency in the distribution of insect-feeding functional modes through time and space, even though the procedures used to define the rank-orders are not identical due to the limitations of fossil data (see Materials and Methods). Abundant DTs in the fossil floras can reflect large populations of few herbivore species as well as a large number of insect species that feed in similar ways.

In the extant plots, low DT similarity among host-plant pairs (Fig. 3A,B; Dataset S1) is strongly related to low compositional similarity for the corresponding feeding insects, suggesting a high degree of host specificity among host plants. On the other hand, high DT similarity among host plants, as in many of the fossil sites, is not informative regarding insect similarity (Fig. 3A–C). This lack of correspondence at high DT similarity is probably associated with the redundancy of feeding traces across herbivore species that arises because many insects overlap in some or most of their leaf-chewing damage. Only those host-plant species pairs having a markedly different DT composition (recorded as low leaf-chewing DT similarity) may be hypothesized to have a comparably low similarity of feeding insects, in living or fossil leaf assemblages (Fig. 3B).

The distributions of species pairwise similarities estimated for fossil assemblages deviate from the null expectation that external DTs are equally distributed across host plant species in a majority of the fossil sites (compare hollow and solid bars in Fig. 3C; see Methods and Table S5), indicating that the observed similarities in fossil assemblages cannot be explained by chance alone. However, within each of the fossil sites, species pairwise similarity estimated from external damage was high overall (>0.8; Fig. 3C and Dataset S1), indicating that little information about host specificity of leaf chewing herbivores is retrievable. It is notable (Fig. 3C) that among the 17 assemblages analyzed, only three floras have conspicuously lower species pairwise similarities (indicating a presumed lower herbivore similarity) than expected by chance (SG, *n* = 13 species, L4H, *n* = 4, and DSt, *n* = 10). These overall lower similarities correspond to three of the four latest Cretaceous floras in the K/Pg boundary interval, contrasting with the overall high similarity values observed in the two post-K/Pg floras. This pattern suggests that host-specificity of leaf-chewing insects also declined after the K/Pg extinction, in addition to endophagous and other specialized, host-specific damage as previously known [2].

The series of floras from the late Paleocene–early Eocene (P–E) interval, Bighorn Basin of Wyoming (Fig. 3C), is uninformative regarding within-site variation in leaf-chewing insect composition. Host-plant DT similarity is overall higher than expected (under the null expectation) in six out of the nine sites from this sequence (Fig. 3C; see Table S5), indicating that the variation of external damage across species within sites is low. This does not alter previous conclusions about significant compositional variation among sites in this sequence in relation to climate change [9], but it does show that the amount of among-site variation for leaf-chewing, in particular, did not change with climate or cannot be detected due to relatively low plant richness at these sites. External damage similarity among host plants is high in the late Paleocene Neotropical rainforest flora from Cerrejón, Colombia, the closest fossil analog to the extant forests examined in Panamá. The high incidence of similar leaf-chewing damage across most host plants in the Cerrejón flora is reflected as minimal host plant specificity, not deviating from the expectation that DTs are randomly distributed across host-plants and consequently rendering no information on culprit similarity. A surprising pattern is found in the early Eocene flora of Laguna del Hunco, where both endophytic and leaf-chewing damage are notably diverse [34], yet similarity of external damage appears slightly higher than our null expectation.

Pairwise similarity of DTs in fossil assemblages has, however, a much wider range when considering all leaf damage, including endophytic feeding such as mines and galls (Fig. 3D; Dataset S1). This effectively increases the signal-to-noise ratio and corroborates patterns that have been previously identified from leaf damage in the fossil record. Observed pairwise similarities show a shift from values lower to higher than expected through the K/Pg transition, in accordance with the ecological perturbation during this interval. In addition, pairwise similarities within the Eocene sites of the Bighorn Basin are mostly lower than expected, better matching the known increase in damage diversity and paleotemperatures, although some of the older late Paleocene sites showed surprisingly low similarities.

## Conclusions

The direct link between richness of leaf-chewing insects and their feeding damage across host plants in two tropical forests validates the underlying assumptions of many paleobiological studies that rely on damage type richness as a means to infer changes in relative herbivore richness through time. Also, the ranked abundances of damage types are nearly identical in modern Panamá and in all fossil assemblages examined, and similarity of insect communities among host plants is detectable from insect damage, particularly when endophytic damage is included. Insect-feeding damage, especially with specialized damage included, is likely to be a robust indicator of relative changes in herbivore diversity and composition in fossil and, of great potential importance, in living forests. For example, given the relative ease of data collection compared to that for live insects, leaf-feeding insect damage would be a promising component of long-term biodiversity monitoring initiatives, especially, as suggested by the fossil record, in areas likely to be affected by climate change and ecological disturbance.

## Supporting Information

Figure S1 **Examples of external damage types (DTs) inflicted by feeding insects of the Área Protegida de San Lorenzo and Parque Natural Metropolitano, described in ref.** [4] **.** (*A*) Hole feeding DT01 inflicted by ‘Chrysomelidae B’ (Coleoptera) on *Luehea seemannii* Triana & Planch (Malvaceae). Sample 08-665. Scale  = 5 mm. (*B*) Hole feeding DT02 (black arrow) inflicted by *Coptocyela leprosa* Boh. (Coleoptera: Chrysomelidae) on *Cordia alliodora* (Ruiz & Pav.) Oken (Boraginaceae). Sample 08-385. Scale 1 cm. (*C*) Hole feeding DT03 inflicted by ‘*Typohorus* sp.’ (Coleoptera: Chrysomelidae) on *Spondias mombin* L. (Anacardiaceae). Sample 08-472. Scale 5 mm. (*D*) Hole feeding DT04 inflicted by ‘*Dicrepidus* sp.’ (Coleoptera: Elateridae) on *Ficus insipida* Willd. (Moraceae). Sample 08-073. (*E*) Hole feeding DT05 inflicted by ‘*Typohorus* sp.’ on *S. mombin*. Sample 08-441. (*F*) Hole feeding DT07 inflicted by ‘Eumolpinae sp1’ (Coleoptera: Chrysomelidae) on *Vochysia ferruginea* Mart. (Vochysiaceae). Sample 09-132. Scale  = 5 mm. (*G*) Hole feeding DT08 inflicted by ‘*Allocolaspis* sp.’ (Coleoptera:Chrysomelidae) on *Cecropia peltata* L. (Urticaceae). Sample 08-450. (*H*) Skeletonization DT21 recorded by ‘*Coptocyela* sp.’ (Coleoptera: Chrysomelidae) on *C. alliodora*. Sample 08-383. (*I*) Margin feeding DT15 inflicted by ‘*Leucothyreus* sp.’ (Coleoptera: Scarabaeidae) on *Pseudobombax septenatum* (Jacq.) Dugand (Malvaceae). Sample 08-344. (*J*) Margin feeding DT12 inflicted by ‘Tettigoniidae sp3’ (Orthoptera) on *Marila laxiflora* Rusby (Calophyllaceae). Sample 09-050. (*K*) Margin feeding DT13 inflicted by ‘Proscopidae sp1’ (Orthoptera) on *Guatteria dumetorum* R.E.Fr (Annonaceae). Sample 09-222. (*L*) Margin feeding DT14 inflicted by ‘Buprestidae sp1’ on *V. ferruginea*. Sample 09-041. (*M*) Skeletonization DT16 inflicted by ‘*Conotrachelus* sp2’ (Coleoptera:Curculionidae) on *Castilla elastica* Sessé (Moraceae). Sample 08-001. (*N*) Skeletonization DT17 inflicted by *Myrmex panamensis* Champion (Coleoptera: Curculionidae) on *C. elastica*. Sample 08-534. (*O*) Margin feeding DT26 inflicted by ‘Tettigoniidae G’ (Orthoptera) on *Anacardium excelsum* (Bertero & Balb. Ex Kunth) Skeels (Anacardiaceae). Sample 08-271. (*P*) Skeletonization DT20 inflicted by *Exophthalmus jekelianus* White (Coleoptera: Curculionidae) on *V. ferruginea*. Sample 09-047. (*Q*) Surface feeding DT31 inflicted by ‘*Apion* sp1’ (Coleoptera: Curculionidae) on leaves of *V. ferruginea*. Sample 09-124. (*R*) Surface feeding DT29 inflicted by ‘*Typohorus* sp.’ (Coleoptera: Chrysomelidae) on *Bonamia trichantha* Hallier f. (Convolvulaceae). Sample 08-581. Scale 5 mm. (*S*) Skeletonization DT24 inflicted by ‘*Compsus* sp.’ (Coleoptera: Curculionidae) on *Vitis tilifolia* (Kunth) Hemsl. (Vitaceae). Sample 08-577. (*T*) Skeletonization DT61 inflicted by ‘*Compsus* sp.’ (Coleoptera: Curculionidae) on *V. tilifolia*. Sample 08-577. (*U*) Hole feeding DT63 inflicted by ‘Tettigoniidae sp5’ (Orthoptera) on *V. ferruginea*. Sample 09-122. (*V*) Margin feeding DT81 inflicted by ‘*Atta* sp.’ (Hymenoptera) on *Jacaranda copaia* (Aubl.) D. Don (Bignoniaceae). Sample 09-154. (*W*) Skeletonization DT22 inflicted by ‘Cryptocephalinae sp1’ (Coleoptera: Chrysomelidae) on leaves of *V. ferruginea*. Sample 09-131. (*X*) Surface feeding DT103 inflicted by ‘Tettigoniidae sp5’ (Orthoptera) on *V. ferruginea*. Sample 09-122. (*Y*) Hole feeding DT68 inflicted by ‘Acrididae sp10’ (Orthoptera) on *G. dumetorum*. Sample 09-221. (*Z*) Hole feeding DT78 inflicted by *Dycladia sp*. (Lepidoptera: Arctiidae) on a leaf of *C. peltata*. Sample 08-114. (*AA*) Surface feeding DT30 inflicted by *Homeolabus analis* Illiger (Coleoptera: Attelabidae) on *T. guianensis*. Sample 09-135. (*BB*) Margin feeding DT143 inflicted by ‘*Steirarrhinus* sp1’ (Coleoptera:Curculionidae) on leaves of *Brosimum utile* (Kunth) Oken (Moraceae). Sample 09-086. (*CC*) Hole feeding DT126 inflicted by ‘Chrysomelidae A’ (Coleoptera) on *A. excelsum*. Sample 08-042.
(TIF)

Figure S2 **External damage types (DTs) recorded by feeding insects of Parque Nacional San Lorenzo and Parque Natural Metropolitano that are not described in ref.** [4] **.** See Table S2 for damage descriptions. (*A*) NDT01. Hole feeding on sample 08-647, *Taeniotes scalaris* Boheman (Coleoptera: Cerambycidae) on *Ficus insipida* Willd. (Moraceae). (*B*) NDT05. Margin feeing on sample 08-178, ‘Tettigoniidae A’ (Orthoptera) on *Luehea seemannii* Triana & Planch. (Malvaceae). (*C*) NDT04. Margin feeding on Sample 08-674, ‘*Atta* sp.’ (Hymenoptera: Formicidae) on *Bonamia tricantha* Hallier f. (Convolvulaceae). (*D*) NDT07. Surface feeding on sample 09-005, ‘Cryptocephalinae sp.20’ (Coleoptera: Chrysomelidae) on *Manilkara bidentata* (A. DC.) A. Chev. (Sapotaceae). (*E*) NDT08. Surface feeding on sample 08-418, *Chersinellina heteropunctuata* Boheman (Coleoptera: Chrysomelidae) on *B. tricantha*. (*F*) NDT02. Hole feeding on sample 08-657, ‘Cerambycidae sp.’ (Coleoptera) on *F. insipida*. (*G*) NDT13. Hole feeding on sample 08-658, *T. scalaris* on *F. insipida* (Moraceae). (*H*) NDT14. Margin feeding on sample 08-673, ‘Curculionidae B’ (Coleoptera) on *L. seemannii*. (*I*) NDT15. Surface feeding on sample 08-290, ‘Curculionidae E’ (Coleoptera) on *L. seemannii*. (*J*) NDT19. Margin feeding on sample 09-190, ‘*Hylobius* sp.’ (Coleoptera: Curculionidae) on *Calophyllum longifolium* Willd. (Calophyllaceae). (*K*) NDT17. Surface feeding on sample 08-150, ‘Chrysomelidae B’ (Coleoptera) on *Spondias mombin* L. (Anacardiaceae). (*L*) NDT20. Margin feeding on sample 09-051, ‘Acrididae sp.20’ (Orthoptera) on *Cordia bicolor* A. DC. (Boraginaceae).
(TIF)

Figure S3 **Relations between recorded damage-types (DTs) and collected insect orders.** Connecting lines represent at least 1 insect species from each order inflicting a given DT. Diameters of DT spheres are scaled relative to the number of insect species recording each DT. Color-code represents preservation index: DTs consistently scored with a preservation index of 1 (white, likely preservation), 0 (black, unlikely preservation), or variably scored as 0 or 1, depending on features of each occurrence (grey).
(TIF)

Figure S4 **Summary of relative frequencies and abundances of damage-types (DTs) in living and fossil forests.** (*A*) Numbers of insect species at Área Protegida de San Lorenzo and Parque Natural Metropolitano (combined) inflicting each DT. The numbers of insect species are shown as the proportion of total insect species, log-transformed for display. (*B*) Relative abundances of leaf-chewing DTs from 17 fossil floras, estimated from 500 randomly selected fossil leaves and repeated 1000 times (see Materials and Methods, and Table S4), log-transformed for display purposes.
(TIF)

Table S1 **Numbers of insect species and families, and their induced damage, collected from 24 host plant species in two tropical forests in Panama.** Twenty insects were subsampled 100 times from each host plant species to obtain mean subsampled richness.
(DOCX)

Table S2 **External damage types recorded by feeding insects of the Área Protegida de San Lorenzo and Parque Natural Metropolitano not described in ref.** [4] **.** See Figure S2.
(DOCX)

Table S3 **Spearman correlation coefficients between IR and DTR at various levels, observed across 24 species of dominant trees and liana species in two Panamanian forests (See** **Fig. 2** **).**
(DOCX)

Table S4 **Comparisons, using Spearman's rank-order correlation coefficients, of damage type relative abundances in fossil leaf assemblages and the observed numbers of culprit species corresponding to damage types in the living forests, using data from 17 Late Cretaceous to middle Eocene fossil sites (see Materials and Methods).** Ranking of modern leaf-chewing DTs is according to the number of insect species observed to record them; ranking of fossil DTs is according to their per-leaf abundances (see Fig. S4; Dataset S1). *P-values* have been adjusted using a Bonferroni correction.
(DOCX)

Table S5 **Number and proportion of observed pairwise similarities not explained under null expectation of DTs being equally distributed across host plant species.**
(DOCX)

Dataset S1 **Description of insect collections and fossil comparisons: Insect collections and recorded leaf damage at Parque Natural Metropolitano and Área Protegida de San Lorenzo, Panamá; Average numbers of DTs recorded at fossil sites, and pairwise similarities of host plant species at modern and fossil sites.**
(XLSX)
