# Supplementary figures and images for: Insect Leaf-Chewing Damage Tracks Herbivore Richness in Modern and Ancient Forests
Source: PLoS One. 2014 May 2;9(5):e94950. doi: 10.1371/journal.pone.0094950 (PMC4008375; doi:10.1371/journal.pone.0094950)

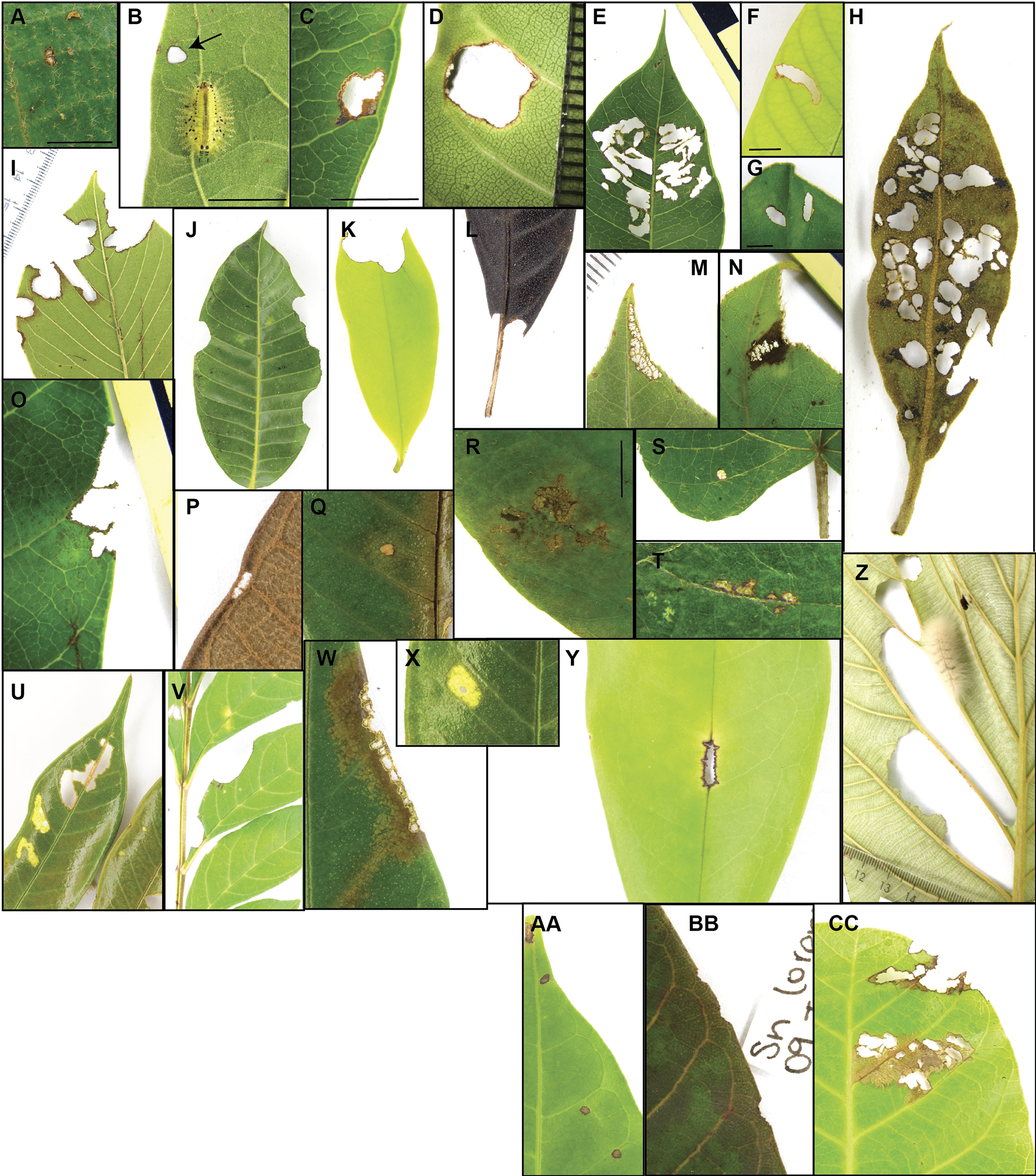

Supplement: Figure S1 — Examples of external damage types (DTs) inflicted by feeding insects of the Área Protegida de San Lorenzo and Parque Natural Metropolitano, described in ref. [4] . (A) Hole feeding DT01 inflicted by ‘Chrysomelidae B’ (Coleoptera) on Luehea seemannii Triana & Planch (Malvaceae). Sample 08-665. Scale = 5 mm. (B) Hole feeding DT02 (black arrow) inflicted by Coptocyela leprosa Boh. (Coleoptera: Chrysomelidae) on Cordia alliodora (Ruiz & Pav.) Oken (Boraginaceae). Sample 08-385. Scale 1 cm. (C) Hole feeding DT03 inflicted by ‘Typohorus sp.’ (Coleoptera: Chrysomelidae) on Spondias mombin L. (Anacardiaceae). Sample 08-472. Scale 5 mm. (D) Hole feeding DT04 inflicted by ‘Dicrepidus sp.’ (Coleoptera: Elateridae) on Ficus insipida Willd. (Moraceae). Sample 08-073. (E) Hole feeding DT05 inflicted by ‘Typohorus sp.’ on S. mombin. Sample 08-441. (F) Hole feeding DT07 inflicted by ‘Eumolpinae sp1’ (Coleoptera: Chrysomelidae) on Vochysia ferruginea Mart. (Vochysiaceae). Sample 09-132. Scale = 5 mm. (G) Hole feeding DT08 inflicted by ‘Allocolaspis sp.’ (Coleoptera:Chrysomelidae) on Cecropia peltata L. (Urticaceae). Sample 08-450. (H) Skeletonization DT21 recorded by ‘Coptocyela sp.’ (Coleoptera: Chrysomelidae) on C. alliodora. Sample 08-383. (I) Margin feeding DT15 inflicted by ‘Leucothyreus sp.’ (Coleoptera: Scarabaeidae) on Pseudobombax septenatum (Jacq.) Dugand (Malvaceae). Sample 08-344. (J) Margin feeding DT12 inflicted by ‘Tettigoniidae sp3’ (Orthoptera) on Marila laxiflora Rusby (Calophyllaceae). Sample 09-050. (K) Margin feeding DT13 inflicted by ‘Proscopidae sp1’ (Orthoptera) on Guatteria dumetorum R.E.Fr (Annonaceae). Sample 09-222. (L) Margin feeding DT14 inflicted by ‘Buprestidae sp1’ on V. ferruginea. Sample 09-041. (M) Skeletonization DT16 inflicted by ‘Conotrachelus sp2’ (Coleoptera:Curculionidae) on Castilla elastica Sessé (Moraceae). Sample 08-001. (N) Skeletonization DT17 inflicted by Myrmex panamensis Champion (Coleoptera: Curculionidae) on C. elastica. Sample 08 [file pone.0094950.s001.tif]

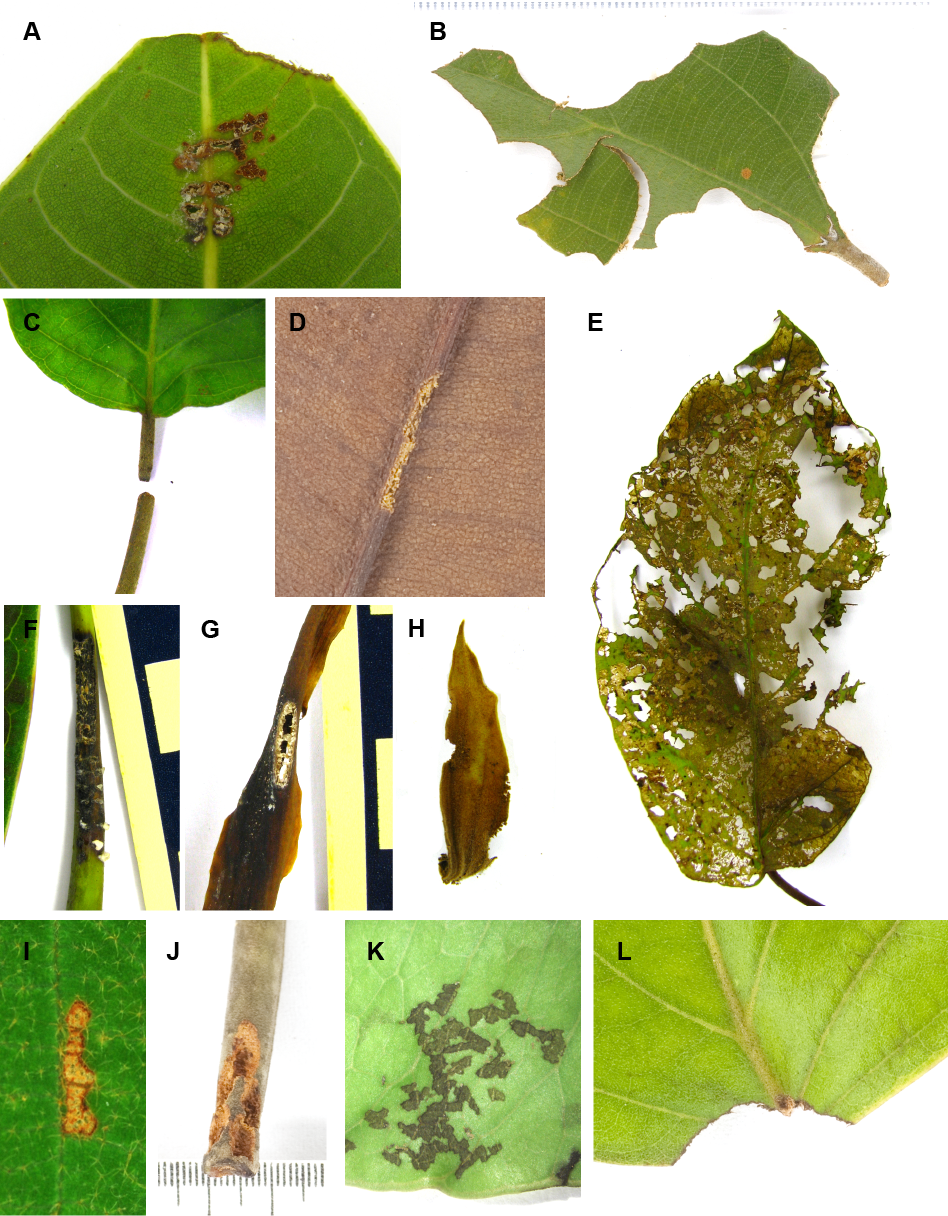

Supplement: Figure S2 — External damage types (DTs) recorded by feeding insects of Parque Nacional San Lorenzo and Parque Natural Metropolitano that are not described in ref. [4] . See Table S2 for damage descriptions. (A) NDT01. Hole feeding on sample 08-647, Taeniotes scalaris Boheman (Coleoptera: Cerambycidae) on Ficus insipida Willd. (Moraceae). (B) NDT05. Margin feeing on sample 08-178, ‘Tettigoniidae A’ (Orthoptera) on Luehea seemannii Triana & Planch. (Malvaceae). (C) NDT04. Margin feeding on Sample 08-674, ‘Atta sp.’ (Hymenoptera: Formicidae) on Bonamia tricantha Hallier f. (Convolvulaceae). (D) NDT07. Surface feeding on sample 09-005, ‘Cryptocephalinae sp.20’ (Coleoptera: Chrysomelidae) on Manilkara bidentata (A. DC.) A. Chev. (Sapotaceae). (E) NDT08. Surface feeding on sample 08-418, Chersinellina heteropunctuata Boheman (Coleoptera: Chrysomelidae) on B. tricantha. (F) NDT02. Hole feeding on sample 08-657, ‘Cerambycidae sp.’ (Coleoptera) on F. insipida. (G) NDT13. Hole feeding on sample 08-658, T. scalaris on F. insipida (Moraceae). (H) NDT14. Margin feeding on sample 08-673, ‘Curculionidae B’ (Coleoptera) on L. seemannii. (I) NDT15. Surface feeding on sample 08-290, ‘Curculionidae E’ (Coleoptera) on L. seemannii. (J) NDT19. Margin feeding on sample 09-190, ‘Hylobius sp.’ (Coleoptera: Curculionidae) on Calophyllum longifolium Willd. (Calophyllaceae). (K) NDT17. Surface feeding on sample 08-150, ‘Chrysomelidae B’ (Coleoptera) on Spondias mombin L. (Anacardiaceae). (L) NDT20. Margin feeding on sample 09-051, ‘Acrididae sp.20’ (Orthoptera) on Cordia bicolor A. DC. (Boraginaceae). (TIF) [file pone.0094950.s002.tif]

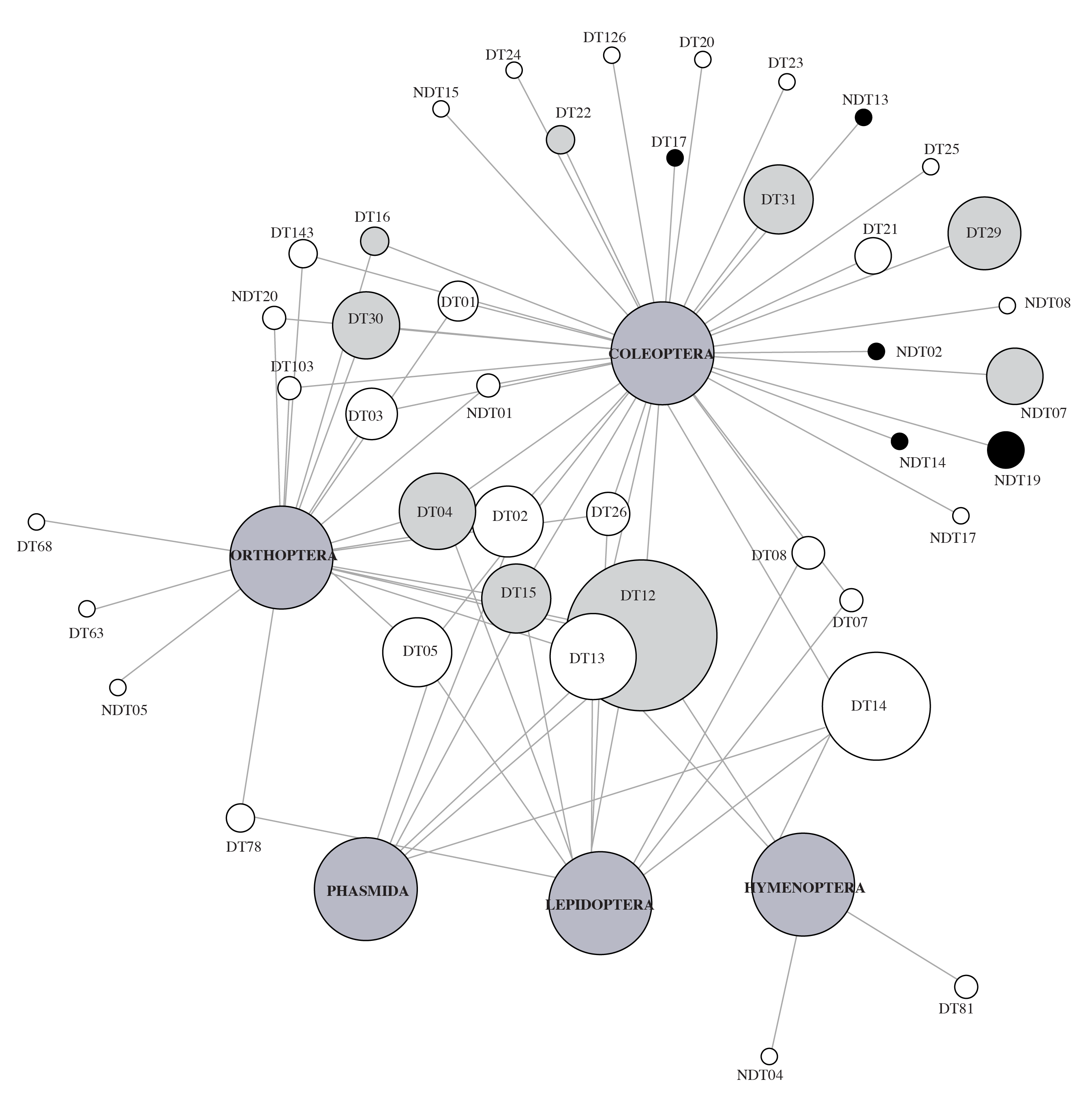

Supplement: Figure S3 — Relations between recorded damage-types (DTs) and collected insect orders. Connecting lines represent at least 1 insect species from each order inflicting a given DT. Diameters of DT spheres are scaled relative to the number of insect species recording each DT. Color-code represents preservation index: DTs consistently scored with a preservation index of 1 (white, likely preservation), 0 (black, unlikely preservation), or variably scored as 0 or 1, depending on features of each occurrence (grey). (TIF) [file pone.0094950.s003.tif]

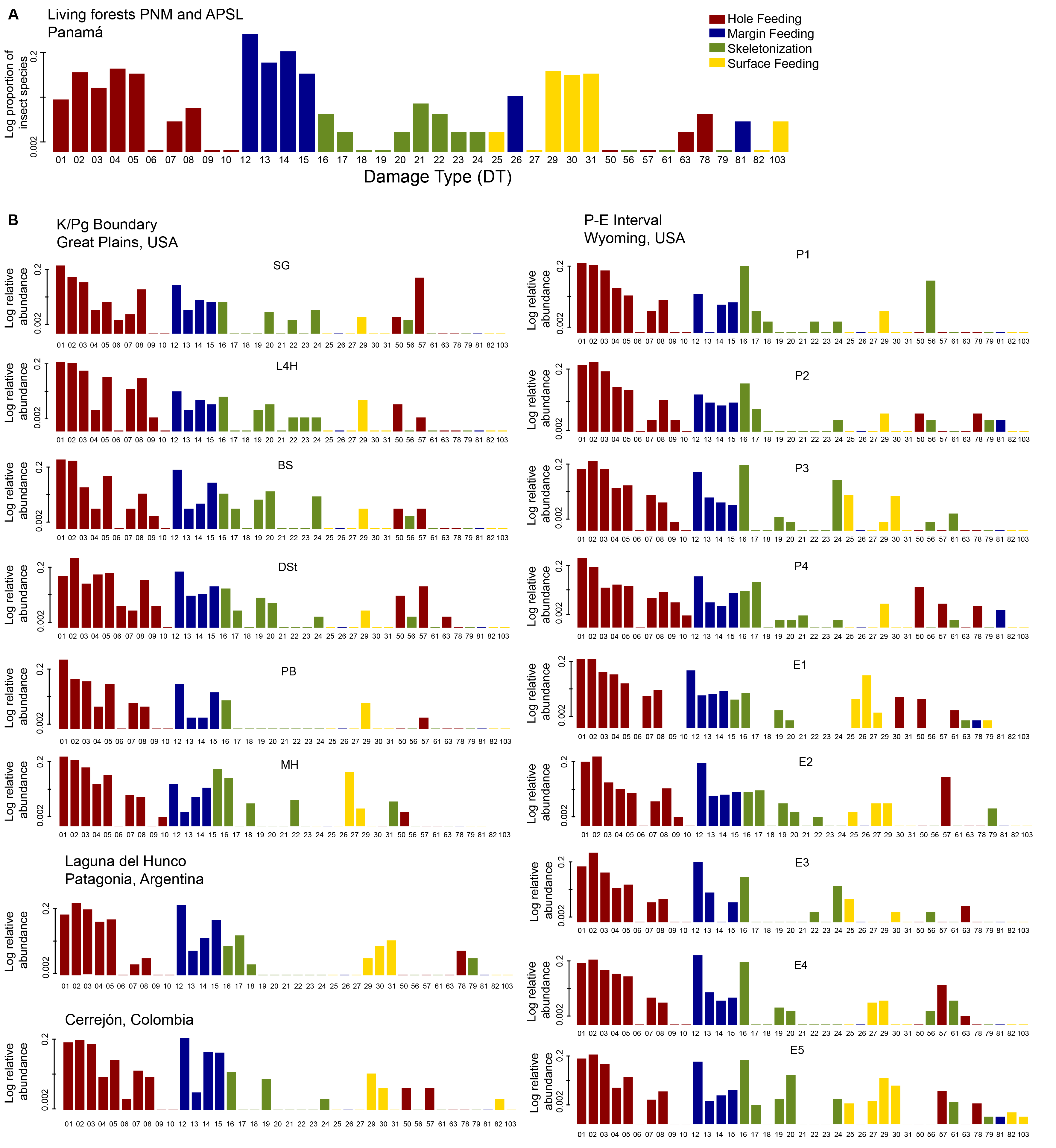

Supplement: Figure S4 — Summary of relative frequencies and abundances of damage-types (DTs) in living and fossil forests. (A) Numbers of insect species at Área Protegida de San Lorenzo and Parque Natural Metropolitano (combined) inflicting each DT. The numbers of insect species are shown as the proportion of total insect species, log-transformed for display. (B) Relative abundances of leaf-chewing DTs from 17 fossil floras, estimated from 500 randomly selected fossil leaves and repeated 1000 times (see Materials and Methods, and Table S4), log-transformed for display purposes. (TIF) [file pone.0094950.s004.tif]
