# Supplementary material for: Insect Leaf-Chewing Damage Tracks Herbivore Richness in Modern and Ancient Forests
Source: PLoS One. 2014 May 2;9(5):e94950. doi: 10.1371/journal.pone.0094950 (PMC4008375; doi:10.1371/journal.pone.0094950)
Supplement: Table S1 — Numbers of insect species and families, and their induced damage, collected from 24 host plant species in two tropical forests in Panama. Twenty insects were subsampled 100 times from each host plant species to obtain mean subsampled richness. (DOCX) [file pone.0094950.s005.docx]

**Table S1. Numbers of insect species and families, and their induced damage, collected from 24 host plant species in two tropical forests in Panama. Twenty insects were subsampled 100 times from each host plant species to obtain mean subsampled richness.**

| Plant Family | Plant Species | Site | Habit | DTR | IR | Family IR | Subsampled DTR | SD | Subsampled IR | SD | Subsampled Family IR | SD |
| --- | --- | --- | --- | --- | --- | --- | --- | --- | --- | --- | --- | --- |
| Anacardiaceae | *Anacardium excelsum* (Kunth) Skeels | PNM | Tree | 14 | 20 | 9 | 11.47 | 1.21 | 12.09 | 1.68 | 6.29 | 1.12 |
| Anacardiaceae | *Astronium graveolens* Jacq. | PNM | Tree | 8 | 7 | 6 | 7.86 | 0.35 | 6.61 | 0.57 | 5.72 | 0.51 |
| Convolvulaceae | *Bonamia trichantha* Hallier f. | PNM | Liana | 11 | 10 | 6 | 9.53 | 1.34 | 8.04 | 1.15 | 4.66 | 1.00 |
| Moraceae | *Castilla elastica* Sessé ex Cerv. | PNM | Tree | 8 | 10 | 7 | 7.52 | 0.67 | 8.56 | 0.96 | 6.32 | 0.69 |
| Cecropiaceae | *Cecropia peltata* L. | PNM | Tree | 10 | 8 | 5 | 9.22 | 0.92 | 7.24 | 0.78 | 4.4 | 0.70 |
| Boraginaceae | *Cordia alliodora* (Ruiz & Pav.) Oken | PNM | Tree | 6 | 5 | 3 | 5.95 | 0.22 | 4.86 | 0.38 | 2.95 | 0.22 |
| Fabaceae | *Enterolobium cyclocarpum* (Jacq.) Griseb. | PNM | Tree | 5 | 9 | 4 | 4.53 | 0.63 | 7.85 | 0.91 | 3.71 | 0.48 |
| Moraceae | *Ficus insipida* Willd. | PNM | Tree | 14 | 18 | 9 | 10.95 | 1.39 | 12.86 | 1.90 | 7.23 | 0.96 |
| Malvaceae | *Luehea seemannii* Triana & Planch. | PNM | Tree | 15 | 17 | 7 | 11.77 | 1.74 | 11.7 | 1.24 | 5.49 | 0.87 |
| Malvaceae | *Pseudobombax septenatum* (Jacq.) Dugand | PNM | Tree | 8 | 9 | 5 | 7.29 | 0.77 | 8.05 | 0.77 | 4.71 | 0.48 |
| Anacardiaceae | *Spondias mombin* L. | PNM | Tree | 6 | 4 | 2 | 5.72 | 0.64 | 3.91 | 0.29 | 1.96 | 0.20 |
| Vitaceae | *Vitis tiliacea* (Kunth) Hemsl. | PNM | Liana | 13 | 6 | 4 | 12.92 | 0.53 | 5.94 | 0.28 | 3.98 | 0.14 |
| Apocynaceae | *Aspidosperma spruceanum* Benth. Ex Müll.Arg. | APSL | Tree | 3 | 2 | 2 | 3* | 0.00 | 2* | 0.00 | 2* | 0.00 |
| Moraceae | *Brosimum utile* (Kunth) Oken ex J. Presl | APSL | Tree | 9 | 11 | 4 | 8.2 | 0.74 | 9.12 | 0.96 | 3.75 | 0.44 |
| Calophyllaceae | *Calophyllum longifolium* Willd. | APSL | Tree | 7 | 8 | 5 | 6.65 | 0.56 | 7.34 | 0.71 | 4.75 | 0.44 |
| Boraginaceae | *Cordia bicolor* A. DC. | APSL | Tree | 3 | 2 | 2 | 3* | 0.00 | 2* | 0.00 | 2* | 0.00 |
| Fabaceae | *Dussia sp.* | APSL | Tree | 5 | 2 | 2 | 5* | 0.00 | 2* | 0.00 | 2* | 0.00 |
| Annonaceae | *Guatteria dumetorum* R.E. Fr. | APSL | Tree | 9 | 15 | 7 | 7.3 | 1.31 | 10.34 | 1.65 | 6.17 | 0.74 |
| Bignoniaceae | *Jacaranda copaia* (Aubl.) D. Don | APSL | Tree | 10 | 7 | 7 | 9.34 | 0.76 | 6.44 | 0.70 | 6.44 | 0.70 |
| Sapotaceae | *Manilkara bidentata* (A. DC.) A. Chev. | APSL | Tree | 7 | 10 | 6 | 6.48 | 0.75 | 8.52 | 0.85 | 5.63 | 0.51 |
| Calophyllaceae | *Marila laxiflora* Rusby | APSL | Tree | 10 | 7 | 6 | 9.42 | 1.72 | 6.76 | 0.45 | 5.78 | 0.44 |
| Anacardiaceae | *Tapirira guianensis* Aubl. | APSL | Tree | 12 | 16 | 6 | 9.26 | 1.17 | 10.03 | 1.72 | 4.07 | 1.02 |
| Combretaceae | *Terminalia amazonia* (J.F. Gmel.) Exell | APSL | Tree | 4 | 6 | 4 | 5.84 | 0.39 | 5.66 | 0.48 | 3.87 | 0.34 |
| Vochysiaceae | *Vochysia ferruginea* Mart. | APSL | Tree | 14 | 11 | 7 | 12.07 | 1.71 | 8.93 | 1.17 | 5.75 | 0.96 |

PNM = Parque Natural Metropolitano; APSL = Área Protegida San Lorenzo. *plants species with collections less than subsampling number.
