# Supplementary material for: Insect Leaf-Chewing Damage Tracks Herbivore Richness in Modern and Ancient Forests
Source: PLoS One. 2014 May 2;9(5):e94950. doi: 10.1371/journal.pone.0094950 (PMC4008375; doi:10.1371/journal.pone.0094950)
Supplement: Table S2 — External damage types recorded by feeding insects of the Área Protegida de San Lorenzo and Parque Natural Metropolitano not described in ref. [4] . See Figure S2. (DOCX) [file pone.0094950.s006.docx]

**Table S2. External damage types recorded by feeding insects of the Área Protegida de San Lorenzo and Parque Natural Metropolitano not described in ref. [4] . See Figure S2.**

| Figure | NDT | External Feeding Category | Description |
| --- | --- | --- | --- |
| S2A | NDT01 | Hole feeding | Linear excision marks cutting transversely through or almost through the midvein. Sample LDP08-647, *Taeniotes scalaris* (Coleoptera:Cerambycidae) on *Ficus insipida* (Moraceae). |
| S2B | NDT05 | Margin feeding | Incomplete curvilinear excision cuts that extend from the leaf margin inwards. Sample LDP08-178, 'Tettigonidae A' (Orthoptera) on *Luehea seemannii* (Malvaceae). |
| S2C | NDT04 | Margin feeding | Complete or nearly complete transversal cutting of leaf petiole. Sample LDP08-674, *Atta sp.* (Hymenoptera:Formicidae) on *Bonamia tricantha* (Convolvulaceae). |
| S2D | NDT07 | Surface feeding | Linear patterns of surface abrasion along the primary veins. Sample LDP09-005, 'Cryptocephalinae sp.20' (Coleoptera:Chrysomelidae) on *Manilkara bidentata* (Sapotaceae). |
| S2E | NDT08 | Surface feeding | Copious surface, hole, and skeletonization feeding throughout the leaf. Sample LDP08-418, *Chersinellina heteropunctuata* (Coleoptera: Chrysomelidae) on *Bonamia tricantha* (Convolvulaceae). |
| S2F | NDT02 | Hole feeding | Series of closely spaced excision marks cutting transversely across unexpanded leaf buds and/or enclosing stipules. Sample LDP08-657, 'Cerambycidae sp.' (Coleoptera) on *Ficus insipida* (Moraceae). |
| S2G | NDT13 | Hole feeding | Chambered elongate holes on stipules showing a distinct thickened reaction rim and a series of leaf tissue projections along its border. Sample LDP08-658, *Taeniotes scalaris* (Coleoptera: Cerambycidae) on *Ficus insipida* (Moraceae). |
| S2H | NDT14 | Margin feeding | Circular, shallow to deep excisions of the stipule margin with less than 180˚ in circumference. Sample LDP08-673, 'Curculionidae B' (Coleoptera) on *Luehea seemannii* (Malvaceae). |
| S2I | NDT15 | Surface feeding | Elongate surface abrasion adjacent to and following secondary or minor secondary veins, and forming one or several expansions and contractions in width along its length. Sample LDP08-290, 'Curculionidae E' (Coleoptera) on *Luehea seemannii* (Malvaceae). |
| S2J | NDT19 | Margin feeding | Excision of segments of the petiole in overlapping series of cusps. Sample LDP09-190, *Hylobius sp.* (Coleoptera:Curculionidae) on *Calophyllum longifolium* (Calophyllaceae). |
| S2K | NDT17 | Surface feeding | Series of overlapping curvilinear, shallow abrasions with a weak reaction rim. Sample LDP08-150, 'Chrysomelidae B' (Coleoptera) on *Spondias mombin* (Anacardiaceae). |
| S2L | NDT20 | Margin feeding | Feeding of the leaf base; excision of the basal-most portion of the leaf blade, resulting in the loss of the petiole, clipped midvein and removed foliar area. Sample LDP09-051, 'Acrididae sp.20' (Orthoptera) on *Cordia bicolor* (Boraginaceae) |
