# Supplementary material for: Insect Leaf-Chewing Damage Tracks Herbivore Richness in Modern and Ancient Forests
Source: PLoS One. 2014 May 2;9(5):e94950. doi: 10.1371/journal.pone.0094950 (PMC4008375; doi:10.1371/journal.pone.0094950)
Supplement: Table S3 — Spearman correlation coefficients between IR and DTR at various levels, observed across 24 species of dominant trees and liana species in two Panamanian forests (See Fig. 2 ). (DOCX) [file pone.0094950.s007.docx]

**Table S3. Spearman correlation coefficients between IR and DTR at various levels, observed across 24 species of dominant trees and liana species in two Panamanian forests (See Fig. 2).**

|  | r_s_ | *P* | n |
| --- | --- | --- | --- |
| IR-DTR | 0.75 | <0.001 | 24 |
| Preservation | 0.71 | <0.001 | 24 |
| Coleoptera | 0.70 | <0.001 | 21 |
| Non coleopteran | 0.51 | 0.01 | 21 |
| Subsampled IR | 0.84 | <0.001 | 24 |
| Family | 0.79 | <0.001 | 24 |
| Monodamagers | 0.98 | <0.001 | 18 |
| Multidamagers | 0.73 | <0.001 | 24 |
