# Supplementary material for: Insect Leaf-Chewing Damage Tracks Herbivore Richness in Modern and Ancient Forests
Source: PLoS One. 2014 May 2;9(5):e94950. doi: 10.1371/journal.pone.0094950 (PMC4008375; doi:10.1371/journal.pone.0094950)
Supplement: Table S4 — Comparisons, using Spearman's rank-order correlation coefficients, of damage type relative abundances in fossil leaf assemblages and the observed numbers of culprit species corresponding to damage types in the living forests, using data from 17 Late Cretaceous to middle Eocene fossil sites (see Materials and Methods). Ranking of modern leaf-chewing DTs is according to the number of insect species observed to record them; ranking of fossil DTs is according to their per-leaf abundances (see Fig. S4; Dataset S1). P-values have been adjusted using a Bonferroni correction. (DOCX) [file pone.0094950.s008.docx]

**Table S4. Comparisons, using Spearman’s rank-order correlation coefficients, of damage type relative abundances in fossil leaf assemblages and the observed numbers of culprit species corresponding to damage types in the living forests, using data from 17 Late Cretaceous to middle Eocene fossil sites (see Materials and Methods). Ranking of modern leaf-chewing DTs is according to the number of insect species observed to record them; ranking of fossil DTs is according to their per-leaf abundances (see Fig. S4; Dataset S1). *P-values* have been adjusted using a Bonferroni correction.**

| Sites compared* | Spearman's rho | *P* | *n* |
| --- | --- | --- | --- |
| Living-SG | 0.49 | 0.02 | 39 |
| Living-L4H | 0.5 | 0.01 | 39 |
| Living-BS | 0.43 | 0.08 | 39 |
| Living-DSt | 0.42 | 0.11 | 39 |
| Living-PB | 0.64 | 0 | 39 |
| Living-MH | 0.53 | 0.008 | 39 |
| Living-P1 | 0.49 | 0.02 | 39 |
| Living-P2 | 0.57 | 0.003 | 39 |
| Living-P3 | 0.52 | 0.01 | 39 |
| Living-P4 | 0.45 | 0.05 | 39 |
| Living-E1 | 0.6 | 0 | 39 |
| Living-E3 | 0.53 | 0.01 | 39 |
| Living-E2 | 0.45 | 0.06 | 39 |
| Living-E4 | 0.47 | 0.03 | 39 |
| Living-E5 | 0.52 | 0.01 | 39 |
| Living-Cerrejón | 0.73 | 0 | 39 |
| Living-LH | 0.55 | 0.005 | 39 |

* **L4H: Luten’s 4H Hadrosaur, SG: Somebody’s Garden, DSt: Dean Street, BS: Battleship, PB: Pyramid Butte, MH: Mexican Hat, P-E: late Paleocene-early Eocene, P1: Skeleton Coast, P2: Lur’d Leaves, P3: Dead Platypus, P4: Daiye Spa, E1: Hubble Bubble, E2: South Fork of Elk Creek, E3: Cool Period, E4: PN, E5: Fifteenmile Creek, LH: Laguna del Hunco.**
