# Supplementary material for: Insect Leaf-Chewing Damage Tracks Herbivore Richness in Modern and Ancient Forests
Source: PLoS One. 2014 May 2;9(5):e94950. doi: 10.1371/journal.pone.0094950 (PMC4008375; doi:10.1371/journal.pone.0094950)
Supplement: Table S5 — Number and proportion of observed pairwise similarities not explained under null expectation of DTs being equally distributed across host plant species. (DOCX) [file pone.0094950.s009.docx]

**Table S5. Number and proportion of observed pairwise similarities not explained under null expectation of DTs being equally distributed across host plant species.**

|  | External Damage | | | Total Damage | | |
| --- | --- | --- | --- | --- | --- | --- |
| Fossil Site* | Similarities lower than null expectation | Similarities greater than null expectation | Proportion not explained by null expectation | Similarities lower than null expectation | Similarities greater than null expectation | Proportion not explained by null expectation |
| SG | 4 | 0 | 0.667 | 18 | 0 | 0.231 |
| L4H | 37 | 0 | 0.474 | 3 | 0 | 0.5 |
| BS | 0 | 0 | 0 | 0 | 1 | 0.022 |
| DSt | 25 | 7 | 0.711 | 0 | 0 | 0 |
| PB | 0 | 0 | 0 | 0 | 0 | 0 |
| MH | 0 | 9 | 0.6 | 0 | 4 | 0.267 |
| P1 | 0 | 0 | 0 | 0 | 0 | 0 |
| P2 | 0 | 10 | 0.476 | 0 | 0 | 0 |
| P3 | 0 | 13 | 0.619 | 9 | 0 | 0.429 |
| P4 | 1 | 9 | 0.476 | 1 | 1 | 0.095 |
| E1 | 0 | 6 | 0.4 | 0 | 1 | 0.067 |
| E3 | 0 | 3 | 0.2 | 2 | 1 | 0.2 |
| E2 | 0 | 0 | 0 | 0 | 0 | 0 |
| E4 | 0 | 0 | 0 | 2 | 0 | 0.667 |
| E5 | 0 | 11 | 0.524 | 17 | 0 | 0.81 |
| LH | 0 | 96 | 0.8 | 0 | 43 | 0.358 |
| Cerrejón | 0 | 1 | 0.067 | 1 | 1 | 0.133 |

* L4H: Luten’s 4H Hadrosaur, SG: Somebody’s Garden, DSt: Dean Street, BS: Battleship, PB: Pyramid Butte, MH: Mexican Hat, P-E: late Paleocene-early Eocene, P1: Skeleton Coast, P2: Lur’d Leaves, P3: Dead Platypus, P4: Daiye Spa, E1: Hubble Bubble, E2: South Fork of Elk Creek, E3: Cool Period, E4: PN, E5: Fifteenmile Creek, LH: Laguna del Hunco.
